# Influence of intestinal microbial metabolites on the abscopal effect after radiation therapy combined with immune checkpoint inhibitors

**DOI:** 10.1016/j.ctro.2024.100758

**Published:** 2024-03-09

**Authors:** Hannah Felchle, Julia Gissibl, Laura Lansink Rotgerink, Sophie M. Nefzger, Caroline N. Walther, Vincent R. Timnik, Stephanie E. Combs, Julius C. Fischer

**Affiliations:** a Department of Radiation Oncology, Klinikum rechts der Isar, TUM School of Medicine and Health, Technical University of Munich, Munich, Germany; b German Cancer Consortium (DKTK), Partner-site Munich and German Cancer Research Center (DKFZ), Heidelberg, Germany; c Helmholtz Zentrum München, Institute of Radiation Medicine, 85764 Neuherberg, Germany

**Keywords:** Abscopal effect, Immune checkpoint inhibition, Intestinal microbiota, Type I Interferon, Bacterial metabolites

## Abstract

**Background:**

Most clinical studies failed to elicit a strong antitumor immune response and subsequent systemic tumor regression after radiation therapy (RT), even in combination with the immune checkpoint inhibitors (ICI) anti-CTLA4 or anti-PD1. Mechanistically, type I interferon (IFN-I) activation is essential for the development of such abscopal effects (AE); however, mechanisms driving or limiting IFN–I activation are ill defined. Groundbreaking discoveries have shown that antibiotics (ABx) can affect oncological outcomes and that microbiota-derived metabolites can modulate systemic antitumor immunity. Recent studies have demonstrated that the bacterial metabolites desaminotyrosine (DAT) and indole-3-carboxaldehyde (ICA) can enhance IFN-I activation in models of inflammatory diseases.

**Materials and Methods:**

The subcutaneous bilateral MC38 tumor model is a widely used experimental tool to study the AE in mice. We applied it to explore the influence of broad-spectrum ABx, DAT and ICA on the AE after radioimmunotherapy (RIT). We performed 1x8 Gy of the primary tumor ± anti-CTLA4 or anti-PD1, and ± daily oral application of ABx or metabolites.

**Result:**

Combinatory ABx had neither a significant effect on tumor growth of the irradiated tumor nor on tumor progression of the abscopal tumor after RIT with anti-CTLA4. Furthermore, DAT and ICA did not significantly impact on the AE after RIT with anti-CTLA4 or anti-PD1. Surprisingly, ICA even appears to reduce outcomes after RIT with anti-CTLA4.

**Conclusion:**

We did not find a significant impact of combinatory ABx on the AE. Experimental application of the IFN-I-inducing metabolites DAT or ICA did not boost the AE after combined RIT. Additional studies are important to further investigate whether the intestinal microbiota or specific microbiota-derived metabolites modulate the AE.

## Highlights

• Intestinal decontamination with broad-spectrum antibiotics has no significant impact on the abscopal effect.
• Oral application of the bacterial metabolite desaminotyrosine (DAT) fails to boost the abscopal effect.
• Oral administration of the bacterial metabolite indole-3-carboxaldehyde (ICA) does not enhance, but tends to suppress, the abscopal effect.

## Introduction

The development of immune checkpoint inhibitors (ICI) revolutionized cancer therapy. In clinical practice, the most commonly used ICIs are antibodies against cytotoxic T lymphocyte antigen 4 (anti-CTLA4) and antibodies blocking the programmed death protein 1 (anti-PD1) or programmed death-ligand 1 (anti-PD-L1) [1]. Among others, systemic treatment approaches using ICIs have raised hope for oligometastatic patients with low metastatic burden previously attributed to palliative treatment regimens [1], [2], [3], [4]. However, there is still a great proportion of patients not responding to ICIs and the mechanisms explaining interindividual differences to ICI therapy are incomplete defined [1], [5]. One strategy to improve outcomes are multimodal treatment regimens combining ICIs with additional therapies such as radiation therapy (RT) and a multitude of pre-clinical studies report the high potential of combined radioimmunotherapy (RIT) [6], [7]. In principle, RT of a single tumor lesion has the potential to induce immunogenic tumor cell death, thereby releasing neoantigens and danger signals (e.g., tumor DNA) which activate antigen presenting cells and induce tumor specific T cell priming. Treatment with ICIs stimulates this T cell priming, and subsequent proliferation and activation of antitumor specific cytotoxic T cells [6], [8]. Experimental and clinical studies have found this process to be highly dependent on the DNA sensing receptor STING (stimulator of interferon genes) which induces type I Interferon (IFN-I) signaling [9], [10], [11], [12], [13]. Primed T cells can then act systemically, targeting the irradiated tumor and unirradiated metastases alike. Regression of unirradiated metastases is called the abscopal effect (AE) [6]. However, even in combination with ICIs, this effect is rarely observed in the clinic and novel strategies to boost the AE are highly investigated [8]. A key study by Vanpouille-Box et al. identified strong IFN-I activation after RT as a prerequisite for the induction of AE in mice and discovered tumor cell intrinsic mechanisms counterbalancing IFN-I activation after RT [9]. Consistently, Formenti et al. found elevated IFN-I blood levels after RT as a predictor for the induction of the AE in patients treated with anti-CTLA4 [13]. However, the mechanisms that control or inhibit IFN-I activation after RT and thus regulate abscopal tumor regression are incompletely understood. Remarkable recent experimental and clinical studies have associated certain gut microbiome profiles with altered systemic treatment responses to chemotherapy and immunotherapy [14], [15], [16], [17] and strategies targeting the intestinal microbiota in the context of immunotherapy (e.g., using fecal microbiota transplantation, FMT) are already investigated in clinical trials [18]. Mechanistically, intestinal microbiome-derived metabolites have been shown to shape antitumor immunity during ICI therapy and microbial compounds are considered potential new therapeutics that improve the clinical efficacy of such anticancer therapies [19], [20]. Recent studies investigating inflammatory diseases linked specific microbiota-derived metabolites to enhanced IFN-I activation. Specifically, oral application of the flavonoid metabolite desaminotyrosine (DAT) and the indole metabolite indole-3-carboxaldehyde (ICA) have been shown to influence immune responses in viral disease and graft-versus-host-disease, respectively [21], [22], [23]. It is therefore explicitly speculated that these metabolites might also immunomodulate response rates to ICI therapy [24]; a speculation that is corroborated by a recent study reporting oral supplementation of DAT to boost cancer immunotherapy with anti-CTLA4 which was dependent on IFN-I signaling in tumor-bearing mice [25]. In contrast, research has just begun exploring how the microbiota modulates antitumor immune responses after RT and even less is understood about a possible influence of the intestinal microbiota on outcomes after combined RIT with ICIs. A first experimental key study by Uribe-Herranz et al. found improved tumor regression after RT in mice treated with Vancomycin [26]. Similarly, Yang et al. found that microbiota-derived butyrate impairs local tumor regression after RT by inhibiting STING activation [27]. In addition, Shiao et al. studied both the bacterial gut microbiota and the intestinal fungome in the context of RT and found a negative impact of the fungome on outcomes after RT [28]. Thus far, no study has analyzed the role of the intestinal microbiota and microbiota–derived metabolites in the modulation of the AE [26], [27], [28]. We here hypothesize that the intestinal bacterial microbiota modulates abscopal response rates to combined RIT and that recently identified IFN–I-inducing metabolites have the potential to boost the AE after RIT.

## Materials and methods

### Mice and animal studies

All animal experiments were ethically evaluated and approved by the local governmental authorities and conducted according to the guidelines to ensure animal welfare (licenses for animal experiments were granted by Regierung von Oberbayern, Munich, Germany). Female C57BL/6J WT mice were purchased at 5 weeks-old from Charles River Laboratories (Research Models and Services, Germany GmbH, Sulzfeld, Baden-Württemberg, Germany) and allowed to acclimate for one week. Mice were housed in individually ventilated cages on a 12 h light–dark cycle and had access to food and water *ad libitum.*

### Tumor cells

All experiments were performed with certified MC38 colon adenocarcinoma cells that were bought from Kerafast (#ENH204-FP) in 2021. Cells were cultured according to standard protocols with DMEM high glucose (#D6429, Sigma-Aldrich), 10 % fetal calf serum, 1 % Penicillin/Streptomycin (10000U/ml) and 1 % HEPES buffer solution (1 M) and continuously tested to be free of mycoplasma. Cell culture prior to tumor induction was standardized and performed with cells of a similar passage (P5-7) after thawing.

### Tumor induction and measurement

Mice were shaved one day before tumor cell injection at the right hind leg and left flank. Tumor cells were injected subcutaneously (s.c.) in a volume of 40 µl PBS and with 1x10^6^ and 5x10^5^ MC38 tumor cells in the right hind leg and the left flank, respectively. Tumor size was determined daily with a caliper measuring the length and width of the tumor. Individual mice were sacrificed when primary or abscopal tumor reached > 300 mm^2^ (length x width) or due to ulceration of any tumor independent of tumor size. For data analysis, tumor volume was calculated with the formula: ½ x (length x width^2^). On day 7 after tumor injection, mice with two tumors were grouped for treatment according to tumor sizes. Extreme outliers in initial tumor size were not included into the analysis. Extreme outliers were defined as a tumor size larger than the mean tumor size of all mice plus 4 standard deviations (>mean + 4 x SD prior to onset of tumor therapy). According to this definition we have excluded n = 4 mice from the experiments which include in total n = 292 mice.

### Radiation therapy of primary tumor (right hind leg)

Mice were anesthetized with an intraperitoneal (i.p.) injection of Medetomidin (0,5 mg/kg), Midazolam (5 mg/kg) and Fentanyl (0,05 mg/kg) and were fixed on a plastic disc before irradiation. Lead plates (3x3 mm = 9 mm; with an estimated reduction of transmission by >99,9%) were used to shield the rest of the body with radiosensitive tissue exposing only the primary tumor bearing right hind leg to the radiation field. Irradiation of the primary tumor with a single dose of 8 Gy was performed using the Gulmay RS225A (Gulmay Medical, Camberley, Surrey, UK) at a dose rate of 0,95 Gy/min (15 mA, 200 kV) or the CIX2 (Xstrahl) at a dose rate of 1,33 Gy/min (15 mA, 195 kV). Confirmation of correct dose rate of the irradiation device was regularly validated. Mice were warmed on a heating pad after irradiation and antagonized with a s.c. injection of Atipamezol (2,5 mg/kg), Flumazenil (0,5 mg/kg) and Naloxon (1,2 mg/kg).

### Immunotherapy with immune checkpoint inhibitors (ICIs)

Indicated experimental groups received i.p. injections with anti-PD-1 (clone RMP1-14, Bio X Cell) and anti-CTLA-4 (clone 9H10, Bio X Cell) in a total volume of 300 µl PBS. MC38 tumor bearing mice were treated with either 100 µg anti-PD-1 or 100 µg anti-CTLA-4 on days 8,11,14,17 and 20 after tumor injection. Control mice were injected with 300 µl PBS.

### Antibiotic treatment

ABx was performed similarly to what others have previously described [29], [30]. Mice were treated with antifungal Amphotericin B (1 mg/kg) twice daily for two days before start of ABx treatment to prevent intestinal fungal overgrowth [31], [32]. Antibiotics were administered in a cocktail of Ampicillin (100 mg/kg), Neomycin (100 mg/kg), Vancomycin (50 mg/kg), Metronidazol (100 mg/kg) and Amphotericin B (1 mg/kg) via 200 µl gavage twice daily (except once daily on weekends) starting 2 days before tumor injection until end of therapy (day 20 after tumor injection).

### Metabolite supplementation

Generally, metabolites were supplied via daily gavage in a volume of 200 µl. Desaminotyrosine (DAT, Sigma H25406-50G) was solved in 1 % DMSO + 2 % Peg400 + H_2_O and Indole-3-Carboxaldehyde (ICA, 129445-5G) in 20 % DMSO + 40 % PEG400 + H_2_O. Mice received DAT (1 mg/day; 2,5mg/day or 5 mg/day) or ICA (1 mg/day) from the day of tumor injection until end of therapy (day 20 after tumor injection). Treatment regimens via oral gavage were adapted for ICA and established for DAT according to the literature [21], [22]. DAT supplementation was once performed via i.p. injections on indicated days as described in detail in the figure legends.

### Statistics

All data are presented as the mean with standard deviation (SD) or standard error of the mean (SEM) as indicated in the individual figure legends. Number of pooled experiments are indicated in the figure legends. GraphPad Prism version 9.3.0 (GraphPad Prism Software, San Diego, California, USA; RRID:SCR_002798) was used for statistical analysis of differences between means of tumor size on day 7 and survival rates of Kaplan-Meier curves. Type of tests are indicated in the figure legends. Longitudinal comparison of tumor growth curves was performed in TumGrowth (https://kroemerlab.shinyapps.io/TumGrowth/) on raw data by two-way ANOVA followed by pairwise comparison between treatment growths until the last day both groups included all experimental animals [33]. Significance was set at p-values < 0.05, p < 0.01 and p < 0.001 (*^,^ ** and ***, respectively). All other p-values values > 0.05 are detailed in the figures or indicated as not significant (n.s.).

## Results

### Intestinal bacterial decontamination does not significantly affect the AE after RIT

**Fig. 1:**
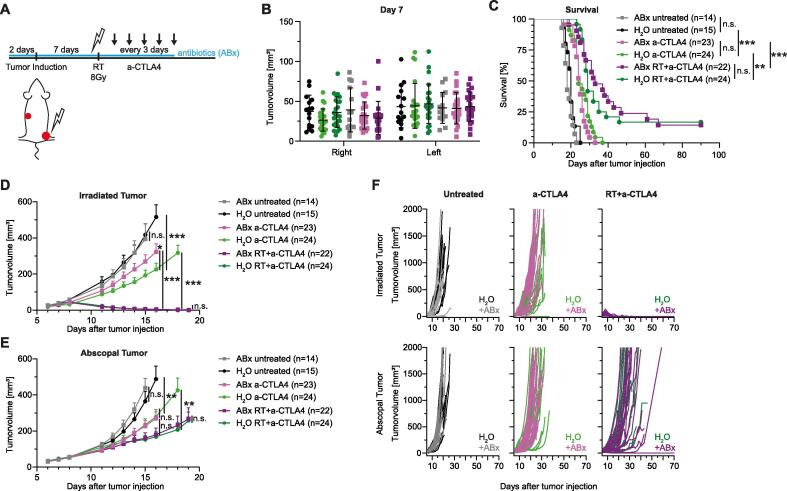
Intestinal bacterial decontamination does not significantly affect the AE after RIT. C57BL/6 wt mice were injected s.c. with MC38 cells (primary tumor: 1x10^6^ cells; abscopal tumor: 5x10^5^ cells) and treated according to the depicted scheme (A). Combination of antibiotics (ABx: 100 mg/kg Ampicillin, 100 mg/kg Neomycin, 50 mg/kg Vancomycin, 100 mg/kg Metronidazole, and 1 mg/kg Amphotericin B) was applied via 200 µl gavage twice daily. ICI with 100 µg anti-CTLA4 was applied i.p.. (B) Stratified groups of tumor sizes on day 7 after tumor induction. Shown is mean ± SD. (C) Kaplan-Meier curves of survival. Statistical comparison was assessed by Log-rank (Mantel-Cox) test. (D) Tumor growth of primary/irradiated tumor. Curves of different treatment groups are depicted until first mouse was taken out of the experiment according to humanized endpoints. Statistical comparison of tumor growth by two-way ANOVA. (E) Tumor growth of secondary/abscopal tumor. Curves of different treatment groups are depicted until first mouse was taken out of the experiment according to humanized endpoints. Statistical comparison of tumor growth by two-way ANOVA. (F) Primary and abscopal tumor growth curves of single mice comparing H_2_O and ABx treated mice with different treatment regimens. All data shown is pooled from 4 independent experiments. Data is presented as mean + SEM if not indicated otherwise. Significance was set at p-values < 0.05, p < 0.01 and p < 0.001 (*^,^ ** and ***, respectively).

Aiming to study microbiota-mediated modulation of the AE, we chose the MC38 tumor model (low immunogenic colorectal tumor cells) considering it is a commonly used experimental model of the AE [34], [35]. First, we combined this model with established antibiotic regimens for intestinal bacterial decontamination to investigate the overall impact of gut microbiota and its bacterial metabolites on AEs [21], [29], [31], [32]. Thus, female C57BL/6 wild type (WT) mice were orally treated with a combination of four antibiotics (ABx) starting two days before MC38 subcutaneous (s.c.) tumor injection and throughout the whole therapy regimen (Fig. 1**A**). As expected, we could not observe any direct influence of oral ABx application on subcutaneous tumor growth after one week of ABx (before starting tumor therapy) **(**Fig. S1**A)**. Next, stratified experimental groups were built before onset of different treatment regimens aiming at balanced initial tumor volumes of individual experimental groups (Fig. 1**B**). Accordingly, untreated mice in control (H_2_O) or ABx treated groups did not differ in tumor growth of primary (Fig. 1**D, F**) or abscopal (Fig. 1**E–F**) tumors, which resulted in comparable overall survival rates and mean survival time (Fig. 1**C**). As expected, H_2_O mice receiving anti-CTLA4 showed significantly reduced tumor growth and significantly prolonged survival compared to H_2_O mice that did not receive anti-CTLA4 (Fig. 1**C–E**). Similarly, we observed significantly enhanced survival and significantly reduced tumor growth in ABx mice treated with anti-CTLA4 as compared to untreated ABx mice (Fig. 1**C–E**). Furthermore, we observed significant reduction of anti-CTLA4 treatment efficacy by ABx only in the primary tumor **(**Fig. 1**D, F)** but not in the abscopal tumor **(**Fig. 1**E, F)**. This effect of tumor growth did not lead to reduced survival in ABx anti-CTLA4 treated mice compared to H_2_O anti-CTLA4 treated mice **(**Fig. 1**C)**. Importantly, survival of mice could be further and significantly improved by combining anti-CTLA4 therapy with additional RT of the primary tumor in ABx and H_2_O mice (Fig. 1**C–F**). Consistently, combined RIT significantly reduced tumor growth at unirradiated/abscopal localization (=AE) and generated long-time survivors with complete tumor regression (Fig. 1**C, E–F**). ABx did not influence treatment response after RIT as compared to H_2_O-treated mice since we did not observe significant differences in abscopal tumor growth (Fig. 1**E–F**) or survival (Fig. 1**C**). Taken together, our data suggests no influence of unspecific intestinal decontamination by continuous ABx on the AE in the MC38 tumor model.

### Desaminotyrosine (DAT) does not significantly boost the AE

**Fig. 2:**
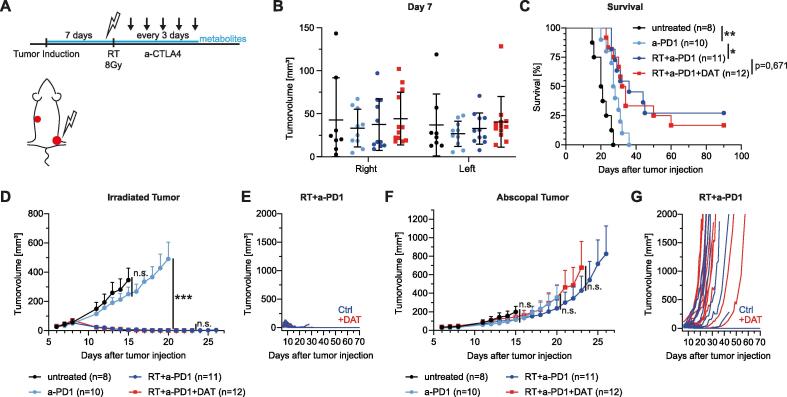
DAT supplementation does not significantly influence AE after RIT with anti-PD1. (A) MC38 tumor bearing mice were subjected to RIT (anti-PD1 100 µg i.p.) and DAT supplementation (1 mg/kg via 200 µl daily gavage). (B) Stratified groups of tumor sizes on day 7 after tumor induction. Shown is mean ± SD. (C) Kaplan-Meier curves of survival. Statistical comparison was assessed by Log-rank (Mantel-Cox) test. (D) Tumor growth of primary/irradiated tumor. Curves of different treatment groups are depicted until first mouse was taken out of the experiment according to humanized endpoints. Statistical comparison of tumor growth by two-way ANOVA. (E) Primary tumor growth curves of single mice comparing RIT treated mice with vehicle or DAT supplementation. (F) Tumor growth of secondary/abscopal tumor. Curves of different treatment groups are depicted until first mouse was taken out of the experiment according to humanized endpoints. Statistical comparison of tumor growth by two-way ANOVA. (G) Abscopal tumor growth curves of single mice comparing RIT treated mice with vehicle or DAT supplementation. All data shown is pooled from 2 independent experiments. Data is presented as mean + SEM if not indicated otherwise. Significance was set at p-values < 0.05, p < 0.01 and p < 0.001 (*^,^ ** and ***, respectively).

**Fig. 3:**
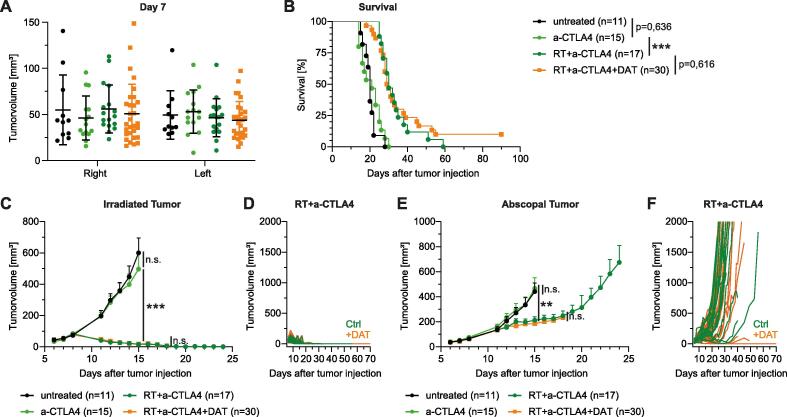
DAT supplementation does not significantly influence the AE after RIT with anit-CTLA4. C57BL/6 wt mice were injected s.c. with MC38 tumor cells and treated with RIT and DAT supplementation as previously described. (A) Stratified groups of tumor sizes on day 7 after tumor. Shown is mean ± SD. (B) Kaplan-Meier curves of survival. Statistical comparison was assessed by Log-rank (Mantel-Cox) test. (C) Tumor growth of primary/irradiated MC38 tumor. Curves of different treatment groups are depicted until first mouse was taken out of the experiment (according to humanized endpoints) except for treatment group with anti-CTLA4 in which 3 mice were taken out of the experiment on day 13 and 14. Statistical comparison of tumor growth by two-way ANOVA. (D) Primary tumor growth curves of single mice comparing RIT treated mice with vehicle or DAT supplementation. (E) Tumor growth of secondary/abscopal tumor. Curves of different treatment groups are depicted until first mouse was taken out of the experiment (according to humanized endpoints) except for treatment group with anti-CTLA4 in which 3 mice were taken out of the experiment on day 13 and 14. Statistical comparison of tumor growth by two-way ANOVA. (F) Abscopal tumor growth curves of single mice comparing RIT treated mice with vehicle or DAT supplementation. All data shown is pooled from 3 independent experiments. To simplify the data comparison, different DAT concentrations (1/2,5/5mg/day) and routes of application (gavage or i.p.) have been pooled into one treatment group. Details are presented in the supplemental date (Fig. S3). Data is presented as mean + SEM if not indicated otherwise. Significance was set at p-values < 0.05, p < 0.01 and p < 0.001 (*^,^ ** and ***, respectively).

Next, we conducted experiments to investigate the therapeutic potential of known IFN-I-inducing microbial metabolites to boost the AE after combined RIT by simultaneous oral application of the metabolites (DAT or ICA) during RIT (Fig. 2**A**). Of note, recent studies had already discovered the IFN-I-inducing potential and the systemic activity of both metabolites after oral application in mice [21], [22]. DAT had no significant effect on tumor growth in the first week after tumor injection (Fig. S2) and treatment groups were stratified on day seven (Fig. 2**B**). We first investigated DAT supplementation in the RIT model with anti-PD1. As expected, RIT led to significantly prolonged survival with long-time survivors (showing clinical complete remission of both tumors) and slightly reduced mean abscopal tumor growth compared to anti-PD1 alone (Fig. 2**C, F**). Strikingly, DAT supplementation did not significantly influence the therapy efficiency as observed in primary and abscopal tumor growth or survival rates (Fig. 2**D–G**). Next, we performed similar experiments with anti-CTLA4. Again, we did not observe any significant modulatory effects of DAT on the AE after RIT (Fig. 3**B–F**). Importantly, exploratory experiments with changed route of administration from orally to intraperitoneally (i.p., Fig. S3) or enhancing the dosage of DAT did not result in different observations. We therefore conclude that DAT supplementation is not able to significantly boost the AE in our experimental mouse model.

### Microbial metabolite ICA appears to negatively affect the AE

**Fig. 4:**
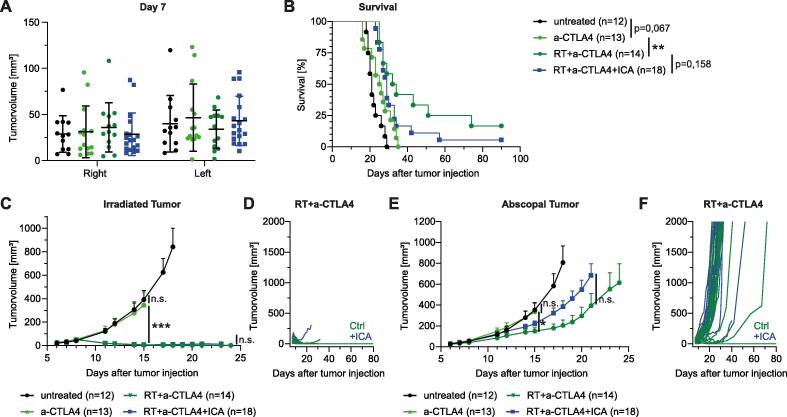
ICA supplementation does also not influence the AE after RIT with a-CTLA4. C57BL/6 wt mice were injected s.c. with MC38 tumor cells and treated with RIT and ICA supplementation (3 mg/day via 200 µl daily gavage). (A) Stratified groups of tumor sizes on day 7 after tumor induction. Shown is mean ± SD. (B) Kaplan-Meier curves of survival. Statistical comparison was assessed by Log-rank (Mantel-Cox) test. (C) Tumor growth of primary/irradiated tumor. Curves of different treatment groups are depicted until first mouse was taken out of the experiment according to humanized endpoints. Statistical comparison of tumor growth by two-way ANOVA. (D) Primary tumor growth curves of single mice comparing RIT treated mice with vehicle or ICA supplementation. (E) Tumor growth of secondary/abscopal tumor. Curves of different treatment groups are depicted until first mouse was taken out of the experiment according to humanized endpoints. Statistical comparison of tumor growth by two-way ANOVA. (F) Abscopal tumor growth curves of single mice comparing RIT treated mice with vehicle or ICA supplementation. All data shown is pooled from 3 independent experiments. Data is presented as mean + SEM if not indicated otherwise. Significance was set at p-values < 0.05, p < 0.01 and p < 0.001 (*^,^ ** and ***, respectively).

Lastly, we investigated a second IFN-I inducing metabolite, namely ICA, on its function to modulate the AE after RIT. We observed that ICA application did not significantly influence tumor growth within the first week after MC38 tumor injection (Fig. S4) and groups were again stratified on day seven (Fig. 4**A**). Surprisingly and contrary to our original hypothesis, we observed a trend that ICA supplementation negatively influences RIT with anti-CTLA4, since additional treatment with ICA reduced overall survival (Fig. 4**B**). Consistently, ICA supplemented mice exhibited increased abscopal tumor growth after RIT with anti-CTLA4 compared to RIT treated mice without ICA (Fig. 4**E, F**). Notably, one mouse receiving RIT and ICA supplementation was unable to control growth of irradiated tumor (Fig. 4**C, D**). Overall, we observed that the recently identified IFN-I-inducing microbial metabolites DAT and ICA are unable to potentiate the AE after RIT and even showed a trend towards the opposite results.

## Discussion

The AE remains a rare phenomenon even when RT is combined with ICIs, and mechanisms that increase and limit systemic response rates to combined RIT are ill defined. Here we speculated that microbiota-derived signals might modulate the AE after RIT and that selected and promising microbiota-derived metabolites might be used to therapeutically boost the AE via enhanced IFN–I activation. This hypothesis was based on recent investigations aiming to elucidate the influence of microbiota-derived signals (e.g., bacterial metabolites) on a functional level to identify possible mechanisms that can be targeted to treat diseases [19], [21], [36], [37], [38]. Remarkable clinical studies found that manipulating patients’ microbiome has great potential not only to ameliorate inflammatory diseases but also to improve cancer therapy [18], [39], [40]. Strikingly, two independent studies found that microbiota-derived metabolites can enhance T cell-driven antitumor immunity during ICI therapy in mice [19], [25]. These discoveries stand in contrast to the rather limited understanding about how the microbiome modulates systemic immune responses after RT and combined RIT [41], [42].

First of all, we found that the combination of four antibiotics (vancomycin, neomycin, ampicillin, metronidazole = VNAM), which is a commonly used regimen for experimental depletion of the murine intestinal microbiota and its metabolites [21], [29], [31], [32], did not significantly impact the AE in mice treated with combined RT and anti-CTLA4. On one hand, this stands in contrast to experimental and clinical studies that found a negative impact of broad spectrum ABx to anti-CTLA4 monotherapy which we were able to partially reproduce using our model with ABx and anti-CTLA4 treated mice [16], [43]. On the other hand, this observation can be interpreted in line with two independent publications reporting that ABx enhanced local response rates and antitumor immunity after monotherapy with RT [26], [27]. Specifically, these studies have shown that vancomycin improves RT efficiency in MC38, B16F1 (melanoma) and B16-OVA (highly immunogenic melanoma) tumor cell bearing mice [26], [27]. Both studies identified reduction of Gram-positive bacteria due to vancomycin as a possible mechanism improving local response rates to RT. Specifically, Yang et al. identified increased circulating butyrate, a Gram-positive bacteria-derived metabolite, to impair the antitumor effects of RT which was mediated by inhibition of STING-dependent IFN-I activation [27]. All in all, supporting literature can be found both for beneficial metabolites (e.g., inosine) associated with enhanced efficacy of ICI monotherapy as well as detrimental metabolites (e.g., butyrate) associated with poorer outcomes after RT monotherapy [19], [26], [27]. By integrating our own data into this context, we speculate that the depletion of all types of bacteria and a subsequent reduction of all metabolites (due to the VNAM gut decontamination regimen) might explain our results of unaltered systemic RIT treatment efficacy in ABx mice. A limitation of our study is therefore that we did not examine the use of individual antibiotics. These aspects could be investigated in further studies. In the second part of our study, we attempted to shift this postulated balance between beneficial and non-beneficial metabolites towards beneficial metabolites through oral supplementation of DAT or ICA. Importantly, both metabolites were already found to have systemic activity and to enhance IFN–I activation after oral application [21], [22]. We hypothesized that such metabolites could boost the AE after RIT via enhanced IFN-I activation. However, our results clearly show that DAT is not sufficient to significantly enhance the AE after RIT with anti-CTLA4 or anti-PD1 in conventionally housed mice. We speculate that there might be sufficient background stimulation of IFN-I-inducing metabolites in mice with a regular (not disrupted) microbiota. This hypothesis is supported by results from Steed et al. that identified DAT in the stool and blood of untreated mice, but not in the stool and only at reduced levels in the blood of ABx-treated dysbiotic mice (of note, Steed et al al. used the same VNAM ABx regimen as utilized in this study) [21]. In line with this assumption, it was demonstrated that an intact microbiota promotes IFN-I activation in intra-tumoral monocytes shaping an antitumorigenic microenvironment and improving antitumor immunity after ICI therapy [44]. However, our findings do not exclude possible beneficial effects of DAT under specific circumstances such as in dysbiotic mice with microbiome changes characterized by reduced amounts of IFN-I activating metabolites (e.g. after treatment with ABx) or in tumor models that differ from ours (e.g. other tumor entities with enhanced immunogenicity). Both speculations are supported by a recent study investigating the effect of DAT supplementation during anti-CTLA4 monotherapy in mice bearing highly immunogenic B16-OVA tumors. Among others, Joachim et al found that (i) oral DAT supplementation improves anti-CTLA4 monotherapy in a IFN-I dependent way and (ii) that oral DAT supplementation compensates for the adverse effects that result from ABx (VNAM) during anti-CTLA4 therapy [25]. Thus, the combination of IFN-I-inducing metabolites with RIT in mice with ABx-induced dysbiosis could be investigated in further studies. Investigating the therapeutic potential of the second metabolite, namely the indole-derivate ICA, we found that this metabolite did not enhance, but instead tended to impair RIT with anti-CTLA4. These results are in contrast with our original hypothesis, and we can only speculate on the underlying mechanisms: it was previously found that application of ICA can impact the composition of the intestinal microbiota during anti-CTLA4 therapy resulting in enhanced bacterial species producing butyrate [45]. As described above, butyrate has been found to negatively affect antitumor immunity by decreasing IFN-I activation after RT monotherapy [27]; thus, we speculate that ICA treatment may have negatively impacted the AE by increasing butyrate. In general, the impact of indoles and tryptophan metabolites on antitumor immunity seems to be highly context dependent. While it was found that the microbial tryptophan metabolite indole-3-aldehyde promotes ICI therapy in murine melanoma via engagement of the aryl hydrocarbon receptor in T cells, activation of AhR signaling in tumor-associated macrophages by microbiota-derived indoles restrained anti-tumor T cell responses in murine models of pancreatic ductal adenocarcinoma [46], [47].

Finally, we would also like to address possible inhibitory factors that could explain our results on metabolite supplementation during RIT. In contrast to acute IFN-I activation during tumor therapy, it is known that excessive and chronic IFN-I activation can lead to contrary effects and inhibits antitumor immunity [48], [49]. Therefore, it is possible that intensive supplementation of IFN-I-inducing metabolites drives such negative effects, and modified treatment regimens with short-term supplementation of IFN-I-inducing metabolites could be investigated in further studies.

In sum, to the best of our knowledge, we here present the first study investigating the role of the intestinal microbiota and specific microbiota-derived bacterial metabolites, during combined RIT. The results are surprising and stand in contrast to our initial hypothesis; however, they are in line with previous radiooncological studies pointing out that the intestinal microbiota modulates response rates to RT in a different manner as compared to monotherapy with ICIs. Our results will help develop further research to identify mechanisms on how to target the gut microbiota or microbiota-derived signaling to improve antitumor immunity after RT and combined RIT.

## Author contributions statement

H.F. established, performed, and analyzed experiments and wrote the manuscript. J.G., L.L.R., C.N.W., S.M.N., and V.R.T helped to perform experiments. S.E.C. provided intellectual input. J.C.F. designed and supervised the study, acquired funding, and wrote the manuscript. All authors received and approved the manuscript. This work is part of the doctoral thesis of H.F. at TUM.

## Conflicts of interests

S.E.C. and J.C.F. received third party funding from the German Research Foundation or the European Union. S.E.C.: Consulting fees from Icotec AG (Switzerland), HMG Systems Engineering GmbH (Germany), Bristol Myers Squibb BMS (Germany). Payment or honoraria for lectures, presentations, speakers bureaus, manuscript writing or educational events (most speaking appointments include reimbursement of travel costs - does not apply for virtual appointments): Roche, BMS, Brainlab, AstraZeneca, Accuray, Dr. Sennewald, Daiichi Sankyo, Elekta, Medac, med update GmbH. All other authors declare that they have no conflicts of interests.

## CRediT authorship contribution statement

**Hannah Felchle:** Data Curation, Writing - Original Draft, Writing - Review & Editing, Visualization, Validation, Formal analysis, Methodology. **Julia Gissibl:** Writing - Review & Editing, Visualization, Methodology. **Laura Lansink Rotgerink:** Writing - Review & Editing, Visualization, Methodology. **Sophie M. Nefzger:** Writing - Review & Editing, Visualization. **Caroline N. Walther:** Writing - Review & Editing, Visualization. **Vincent R. Timnik:** Writing - Review & Editing, Visualization. **Stephanie E. Combs:** Writing - Review & Editing, Resources. **Julius C. Fischer:** Conceptualization, Funding acquisition, Data Curation, Writing - Original Draft, Writing - Review & Editing, Validation, Methodology, Supervision, Project administration.

## Declaration of Competing Interest

The authors declare that they have no known competing financial interests or personal relationships that could have appeared to influence the work reported in this paper.

## References

[1] Bagchi S., Yuan R., Engleman E.G. (2021). Immune Checkpoint Inhibitors for the Treatment of Cancer: Clinical Impact and Mechanisms of Response and Resistance. Annu Rev Pathol.

[2] Theelen W. (2019). *Effect of Pembrolizumab After Stereotactic Body Radiotherapy vs Pembrolizumab Alone on Tumor Response in Patients With Advanced Non-Small Cell Lung Cancer: Results of the PEMBRO-RT Phase 2 Randomized Clinical Trial.* JAMA. Oncol.

[3] Theelen W. (2021). Pembrolizumab with or without radiotherapy for metastatic non-small-cell lung cancer: a pooled analysis of two randomised trials. Lancet Respir Med.

[4] Palma D.A. (2019). Stereotactic ablative radiotherapy versus standard of care palliative treatment in patients with oligometastatic cancers (SABR-COMET): a randomised, phase 2, open-label trial. Lancet.

[5] Morad G. (2021). Hallmarks of response, resistance, and toxicity to immune checkpoint blockade. Cell.

[6] Ngwa W. (2018). Using immunotherapy to boost the abscopal effect. Nat Rev Cancer.

[7] Jagodinsky J.C., Harari P.M., Morris Z.S. (2020). The Promise of Combining Radiation Therapy With Immunotherapy. Int J Radiat Oncol Biol Phys.

[8] Galluzzi L. (2023). Emerging evidence for adapting radiotherapy to immunotherapy. Nat Rev Clin Oncol.

[9] Vanpouille-Box C. (2017). DNA exonuclease Trex1 regulates radiotherapy-induced tumour immunogenicity. Nat Commun.

[10] Burnette B.C. (2011). The efficacy of radiotherapy relies upon induction of type i interferon-dependent innate and adaptive immunity. Cancer Res.

[11] Harding S.M. (2017). Mitotic progression following DNA damage enables pattern recognition within micronuclei. Nature.

[12] Deng L. (2014). STING-Dependent Cytosolic DNA Sensing Promotes Radiation-Induced Type I Interferon-Dependent Antitumor Immunity in Immunogenic Tumors. Immunity.

[13] Formenti S.C. (2018). Radiotherapy induces responses of lung cancer to CTLA-4 blockade. Nat Med.

[14] Matson V. (2018). The commensal microbiome is associated with anti-PD-1 efficacy in metastatic melanoma patients. Science.

[15] Gopalakrishnan V. (2018). Gut microbiome modulates response to anti-PD-1 immunotherapy in melanoma patients. Science.

[16] Vetizou M. (2015). Anticancer immunotherapy by CTLA-4 blockade relies on the gut microbiota. Science.

[17] Viaud S. (2013). The intestinal microbiota modulates the anticancer immune effects of cyclophosphamide. Science.

[18] Davar D. (2021). Fecal microbiota transplant overcomes resistance to anti-PD-1 therapy in melanoma patients. Science.

[19] Mager L.F. (2020). Microbiome-derived inosine modulates response to checkpoint inhibitor immunotherapy. Science.

[20] Allen-Vercoe E., Coburn B. (2020). A Microbiota-Derived Metabolite Augments Cancer Immunotherapy Responses in Mice. Cancer Cell.

[21] Steed A.L. (2017). The microbial metabolite desaminotyrosine protects from influenza through type I interferon. Science.

[22] Swimm A. (2018). Indoles derived from intestinal microbiota act via type I interferon signaling to limit graft-versus-host disease. Blood.

[23] Thiele Orberg E. (2024). Bacteria and bacteriophage consortia are associated with protective intestinal metabolites in patients receiving stem cell transplantation. Nat Cancer.

[24] Zitvogel L. (2018). The microbiome in cancer immunotherapy: Diagnostic tools and therapeutic strategies. Science.

[25] Joachim L. (2023). The microbial metabolite desaminotyrosine enhances T-cell priming and cancer immunotherapy with immune checkpoint inhibitors. EBioMedicine.

[26] Uribe-Herranz M. (2020). Gut microbiota modulate dendritic cell antigen presentation and radiotherapy-induced antitumor immune response. J Clin Invest.

[27] Yang K. (2021). Suppression of local type I interferon by gut microbiota-derived butyrate impairs antitumor effects of ionizing radiation. J Exp Med.

[28] Shiao S.L. (2021). Commensal bacteria and fungi differentially regulate tumor responses to radiation therapy. Cancer Cell.

[29] Rakoff-Nahoum S. (2004). Recognition of commensal microflora by toll-like receptors is required for intestinal homeostasis. Cell.

[30] Fischer J.C. (2017). Assessment of mucosal integrity by quantifying neutrophil granulocyte influx in murine models of acute intestinal injury. Cell Immunol.

[31] Reikvam D.H. (2011). Depletion of murine intestinal microbiota: effects on gut mucosa and epithelial gene expression. PLoS One.

[32] Tirelle P. (2020). Comparison of different modes of antibiotic delivery on gut microbiota depletion efficiency and body composition in mouse. BMC Microbiol.

[33] Enot D.P. (2018). TumGrowth: An open-access web tool for the statistical analysis of tumor growth curves. Oncoimmunology.

[34] Brix N. (2017). Abscopal, immunological effects of radiotherapy: Narrowing the gap between clinical and preclinical experiences. Immunol Rev.

[35] Wei J. (2021). Sequence of alphaPD-1 relative to local tumor irradiation determines the induction of abscopal antitumor immune responses. Sci Immunol.

[36] Winkler E.S. (2020). The Intestinal Microbiome Restricts Alphavirus Infection and Dissemination through a Bile Acid-Type I IFN Signaling Axis. Cell.

[37] Guo H. (2020). Multi-omics analyses of radiation survivors identify radioprotective microbes and metabolites. Science.

[38] Stein-Thoeringer C.K. (2019). Lactose drives Enterococcus expansion to promote graft-versus-host disease. Science.

[39] van Lier Y.F. (2020). Donor fecal microbiota transplantation ameliorates intestinal graft-versus-host disease in allogeneic hematopoietic cell transplant recipients. Sci Transl Med.

[40] Baunwall S.M.D. (2020). Faecal microbiota transplantation for recurrent Clostridioides difficile infection: An updated systematic review and meta-analysis. EClinicalMedicine.

[41] Roy S., Trinchieri G. (2017). Microbiota: a key orchestrator of cancer therapy. Nat Rev Cancer.

[42] Cytlak U.M. (2021). Immunomodulation by radiotherapy in tumour control and normal tissue toxicity. Nat Rev Immunol.

[43] Chaput N. (2019). Baseline gut microbiota predicts clinical response and colitis in metastatic melanoma patients treated with ipilimumab. Ann Oncol.

[44] Lam K.C. (2021). Microbiota triggers STING-type I IFN-dependent monocyte reprogramming of the tumor microenvironment. Cell.

[45] Renga G. (2022). Optimizing therapeutic outcomes of immune checkpoint blockade by a microbial tryptophan metabolite. J Immunother Cancer.

[46] Bender M.J. (2023). Dietary tryptophan metabolite released by intratumoral Lactobacillus reuteri facilitates immune checkpoint inhibitor treatment. Cell.

[47] Hezaveh K. (2022). Tryptophan-derived microbial metabolites activate the aryl hydrocarbon receptor in tumor-associated macrophages to suppress anti-tumor immunity. Immunity.

[48] Chen J. (2019). Type I IFN protects cancer cells from CD8+ T cell-mediated cytotoxicity after radiation. J Clin Invest.

[49] Jacquelot N. (2019). Sustained Type I interferon signaling as a mechanism of resistance to PD-1 blockade. Cell Res.

