# Supplementary material for: Influence of intestinal microbial metabolites on the abscopal effect after radiation therapy combined with immune checkpoint inhibitors
Source: Clin Transl Radiat Oncol. 2024 Mar 9;46:100758. doi: 10.1016/j.ctro.2024.100758 (PMC10945164; doi:10.1016/j.ctro.2024.100758)
Supplement: Supplementary data 1 [file mmc1.pdf]

Supplemental Material

Influence of intestinal microbial metabolites on the abscopal effect after radiation therapy combined with immune checkpoint inhibitors

Hannah Felchle, Julia Gissibl, Laura Lansink Rotgerink, Sophie M. Nefzger, Caroline N. Walther, Vincent R. Timnik, Stephanie E. Combs, and Julius C. Fischer

Contents

- Supplemental Figures S1-S4

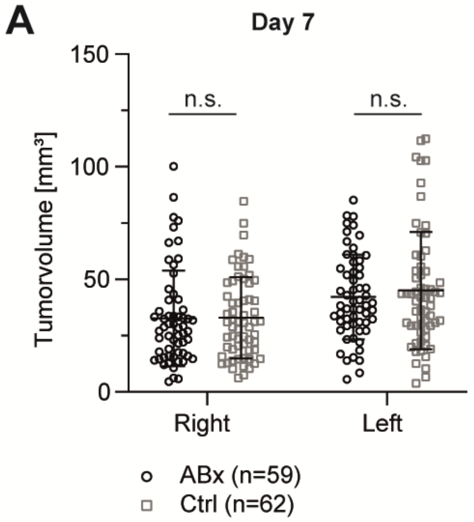

Figure S1

Figure S1: ABx treatment has no effect on tumor growth until start of therapy regimen.

A) MC38 tumor size on day seven after tumor induction and ABx treatment. Data is shown as mean ± SD. Statistical comparison by one-way ANOVA with Tukey’s multiple comparisons.

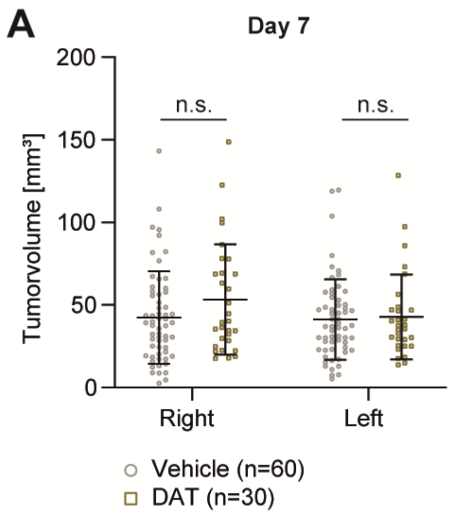

Figure S2

Figure S2: DAT supplementation has no effect on tumor growth until start of therapy regimen.

A) MC38 tumor size on day seven after tumor induction and DAT supplementation (here 1mg/day). Data is shown as mean ± SD. Statistical comparison by one-way ANOVA with Tukey's multiple comparisons.

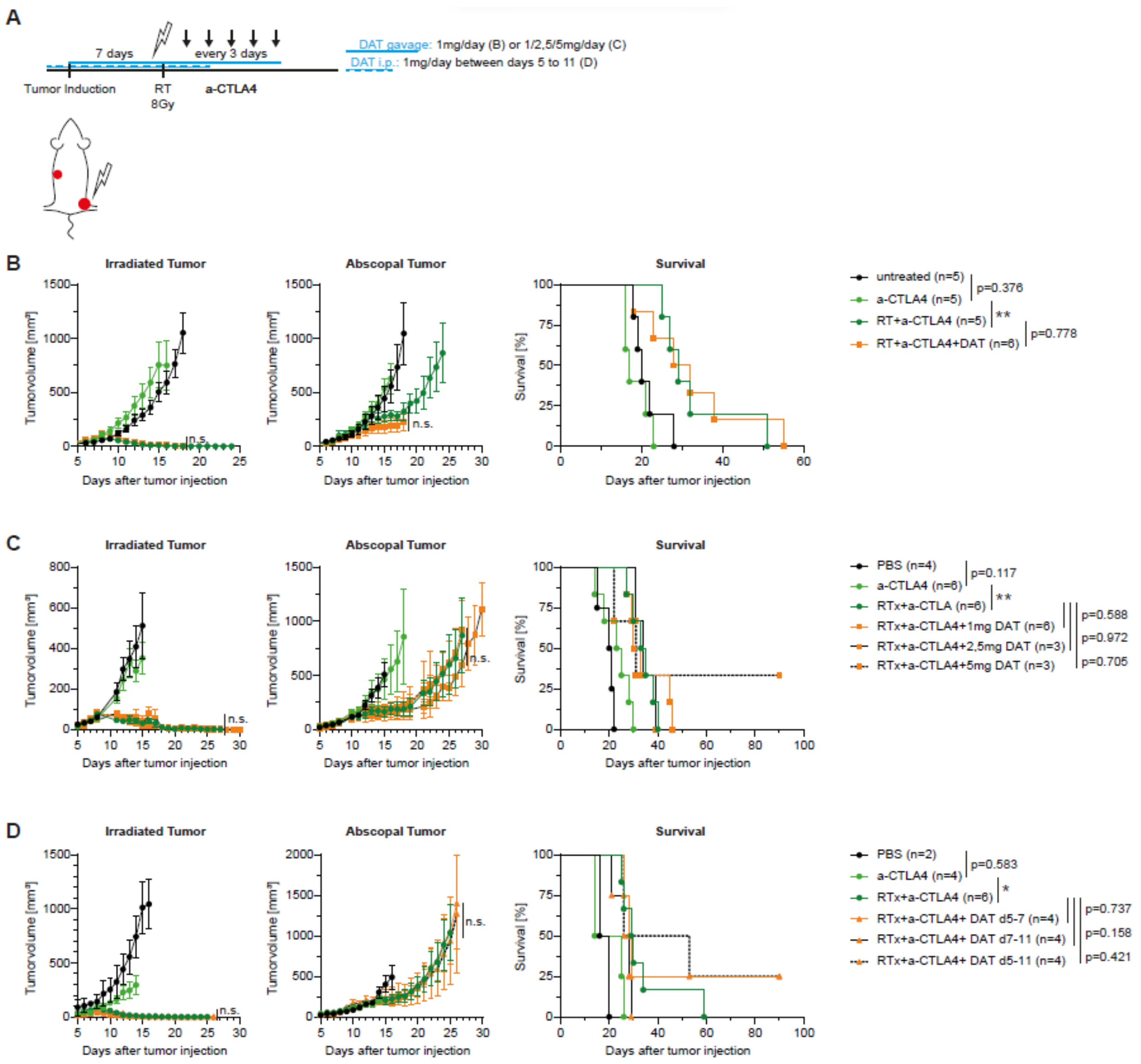

Figure S3: Enhanced dosage of DAT or changed route of application does not induce any effects on AE.

(A) MC38 tumor bearing mice were subjected to RIT (8Gy + anti-CTLA4 100µg i.p.) and DAT supplementation via indicated different routes of application and time intervals (gavage (B,C) versus i.p. (D)), or enhanced dosages by daily gavage. **(B)** Tumor growth of primary/irradiated and secondary/abscopal MC38 tumor after 1mg/day DAT daily gavage. Curves of different treatment groups are depicted until first mouse was taken out of the experiment according to humanized endpoints. Statistical comparison of tumor growth by two-way ANOVA. Kaplan-Meier curves of survival. Statistical comparison of survival was assessed by Log-rank (Mantel-Cox) test. Data shown is from one individual experiment. **(C)** Tumor growth of primary/irradiated and

secondary/abscopal MC38 tumor after 1 or 2,5 or 5mg/day DAT daily gavage. Curves of different treatment groups are depicted until first mouse was taken out of the experiment (according to humanized endpoints) except for treatment group with anti-CTLA4 in which one mouse was taken out of the experiment on day 13. Statistical comparison of tumor growth by two-way ANOVA. Kaplan-Meier curves of survival. Statistical comparison of survival was assessed by Log-rank (Mantel-Cox) test. Data shown is from one individual experiment. **(D)** Tumor growth of primary/irradiated and secondary/abscopal MC38 tumor after DAT 1mg/day by i.p. injection on indicated days. Application vehicle was 1%DMSO in PBS. Curves of different treatment groups are depicted until first mouse was taken out of the experiment according to humanized endpoints. Statistical comparison of tumor growth by two-way ANOVA. Kaplan-Meier curves of survival. Statistical comparison of survival was assessed by Log-rank (Mantel-Cox) test. Data is from one individual experiment. All data is presented as mean + SEM if not indicated otherwise. Significance was set at p-values <0.05, $p < 0.01$ and $p < 0.001$ (*, ** and ***, respectively).

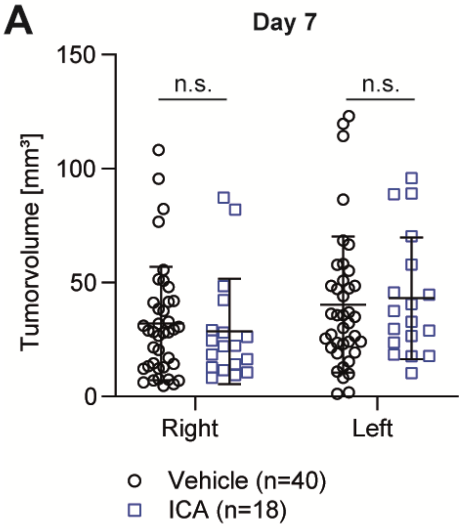

Figure S4

Figure S4: ICA supplementation has no effect on tumor growth until start of therapy regimen.

A) MC38 tumor size on day seven after tumor induction and ICA supplementation. Data is shown as mean ± SD. Statistical comparison by one-way ANOVA with Tukey's multiple comparisons.
